# Artificial Intelligence Performance in Cardiac Magnetic Resonance Strain Analysis for Aortic Stenosis: Validation with Echocardiography and Healthy Controls

**DOI:** 10.3390/medicina61060950

**Published:** 2025-05-22

**Authors:** Žygimantas Abramikas, Ieva Jasiukevičiūtė, Giedrė Balčiūnaitė, Sigita Glaveckaitė, Darius Palionis, Nomeda Valevičienė

**Affiliations:** 1 Clinic of Cardiovascular Diseases, Institute of Clinical Medicine, Faculty of Medicine, Vilnius University, LT-03101 Vilnius, Lithuaniasigita.glaveckaite@santa.lt (S.G.); 2 Faculty of Medicine, Vilnius University, LT-03101 Vilnius, Lithuania; 3 Vilnius University Hospital Santaros Klinikos, LT-03101 Vilnius, Lithuania; darius.palionis@santa.lt (D.P.); nomeda.valeviciene@santa.lt (N.V.)

**Keywords:** aortic stenosis, cardiac MRI, artificial intelligence, global longitudinal strain, echocardiography, myocardial strain

## Abstract

*Background and Objectives*: Aortic stenosis (AS) leads to progressive left ventricular (LV) dysfunction, making early detection crucial. Global longitudinal strain (GLS) is an echocardiographic marker of subclinical LV dysfunction; however, echocardiography has limitations, including operator dependency and acoustic variability. Cardiac magnetic resonance (CMR) is a valuable complementary tool, and artificial intelligence (AI) may enhance strain measurement accuracy, though its role in AS remains underexplored. To evaluate the performance of an AI-based CMR feature tracking tool for the assessment of LV global and segmental GLS in AS patients and compare results with the respective measurements from healthy volunteers (control group), as well as with the GLS obtained using the echocardiographic speckle tracking technique. *Materials and Methods*: This retrospective study analysed 111 CMR exams (70 AS patients, 41 healthy controls) from a single centre. AI-derived GLS values from gradient echo 2-, 3-, and 4-chamber CMR views were manually reviewed for accuracy. Error rates, segmental, and global myocardial strain differences were assessed between AS patients and the control group. *Results*: AI-based CMR GLS strongly correlated with echocardiographic GLS (r = 0.694, *p* < 0.001) and showed lower variability. The AI-derived GLS from CMR was significantly lower in aortic stenosis patients compared to controls (−17.86 ± 3.47 vs. −20.70 ± 1.98). However, AI-based strain analysis had an overall error rate of 6%, which was significantly higher in AS patients (18.6%) compared to healthy controls (2.44%) (*p* = 0.0088). The 3-chamber CMR view was the most error-prone (50% of isolated errors). Segmental strain variability between AS patients and controls was most pronounced in basal segments, with smaller differences in middle and apical segments. CMR demonstrated greater precision than echocardiography, as indicated by a smaller standard deviation in GLS measurements (3.47 vs. 4.98). *Conclusions*: The AI-based CMR feature tracking technique provides accurate and reproducible GLS measurements, showing strong agreement with echocardiographic speckle tracking-based GLS. However, the higher error rates in AS patients compared to controls underscore the need for more advanced AI algorithms to improve performance in cardiac pathology.

## 1. Introduction

Aortic stenosis (AS) is the most common acquired valvular heart disease. The prevalence of AS increases with age, from 0.2% in individuals aged 50–59 years to 9.8% in those aged 80–89 years. The rise in incidence is expected in the next decades with the ageing of the population [1,2]. The subclinical phase of AS is asymptomatic and does not affect mortality. However, once symptoms like angina, syncope or heart failure appear, untreated patients experience a notable decline in average survival, with a prognosis of 2 to 5 years without intervention [3,4]. Early detection and intervention, however, are associated with reduced all-cause, cardiovascular and non-cardiovascular mortality without an increase in any procedure-related clinical outcomes among asymptomatic severe AS patients [5]. That is why imaging plays a huge role in the timely diagnosis, assessment of severity and guiding interventions. Echocardiography is essential for confirming the diagnosis and severity of AS, evaluating valve calcification, left ventricular function and wall thickness, identifying concomitant valve diseases or aortic abnormalities and providing prognostic information [6]. Echocardiography, however, has limitations such as poor acoustic windows and operator dependency, which can make accurate quantification of AS difficult, especially when discordant results among different stenotic indices are observed. CMR, on the other hand, provides both a reliable and reproducible alternative for assessing the aortic valve morphology and function, as well as structural and functional information of the LV in AS patients [7]. Compared to transthoracic echocardiography (TTE), CMR provides more precise assessments of LV volume, function and remodelling. Additionally, it enables the detection of myocardial fibrosis—a critical factor in AS progression. Late gadolinium enhancement (LGE) allows for the visualisation of focal myocardial scarring, which is associated with adverse clinical outcomes, whereas T1 mapping facilitates the quantification of diffuse interstitial fibrosis. CMR-based GLS analysis can also detect subclinical myocardial dysfunction even in patients with preserved ejection fraction. Impaired GLS has been correlated with AS progression and poor prognosis, highlighting its potential in risk stratification and treatment planning [8]. As AI-driven methods continue to advance, automated CMR-based strain analysis could further enhance diagnostic precision and improve clinical decision-making. Recent advancements in artificial intelligence, especially deep learning, have further transformed CMR. AI automates post-processing, enhances diagnostic accuracy and streamlines workflows by reducing operator variability and improving image quality [9]. Given these advancements, AI-based approaches have gained increasing interest in cardiovascular imaging, particularly in the quantitative assessment of myocardial function. This study aims to evaluate the performance of an AI-based CMR feature tracking tool for the assessment of LV global and segmental GLS in AS patients and compare results with the respective measurements from healthy volunteers (control group), as well as with the GLS obtained using the echocardiographic speckle tracking technique.

## 2. Materials and Methods

### 2.1. Study Population

A total of 111 CMR cases were analysed in this study, 70 of severe AS patients before surgical aortic valve replacement (precise patient cohort description can be found in [10]) and 41 of healthy controls. Patients included in this study were diagnosed with AS based on echocardiographic criteria, following the guidelines of the European Society of Cardiology [6]. Patients with standard contraindications for CMR were excluded. This study was approved by the local biomedical research ethics committee (number: 158200-18/9-1014-558, 4 September 2018; number 2023/7-1532-990, 4 July 2023; Vilnius Regional Biomedical Research Ethics Committee) and conformed to the principles of the Helsinki Declaration.

### 2.2. Imaging Protocols

CMR scans were performed at the Vilnius University Hospital Santaros Klinikos from November 2018 to March 2024 using 1.5 T magnetic resonance scanners (either Philips Ingenia Ambition (Eindhoven, The Netherlands) or Siemens Aera (Erlangen, Germany). Three standard imaging views were captured: gradient echo cine 2-chamber, 3-chamber, and 4-chamber.

Automated GLS measurements were analysed using SuiteHEART, Version 5.1.1 (Neosoft, Pewaukee, WI, USA). These analyses were fully automated, but all results were manually reviewed for accuracy.

A trained research assistant (a medical student supervised by a radiologist) manually corrected the epicardial and endocardial contours in cases where the AI tool failed to accurately analyse these structures. After ensuring all cases were properly prepared, segmental analysis of myocardial strain between patients with aortic stenosis and healthy controls was conducted.

Echocardiography was performed using a Vivid ultrasound system (model S70, E9, or E95, GE Healthcare, Horten, Norway) in patients with severe AS before surgical aortic valve replacement and served as the reference standard for GLS measurements. From the 2D grey-scale images of the apical 2-, 3-, and 4-chamber views, LV GLS was measured and processed off-line using commercially available software (EchoPac 112.0.1, GE Medical Systems, Horten, Norway). The frame rate was adjusted to 50 to 80 frames/s. End-systole was defined based on the closure click on the spectral tracing of the pulsed-wave Doppler of AV flow. GLS was acquired using the average regional strain curves 16-segment model for 2D speckle tracking echocardiography. Segments with poor quality tracking or aberrant curves (despite manual adjustment) were removed from the analysis.

### 2.3. Data Analysis

Fisher’s exact test was applied to determine whether there is an association between having AS and CMR AI’s interpretation needing manual corrections. The normality of the GLS was checked by using a Kolmogorov–Smirnov test due to the sample size being >50. After ensuring all cases were properly prepared, segmental analysis of myocardial strain between patients with AS and healthy controls was conducted using Student’s *t*-test. The threshold for statistical significance was set to *p* < 0.05. All statistical analyses were performed using IBM SPSS Statistics, Version 27 (IBM Corp., Armonk, NY, USA).

## 3. Results

### 3.1. Performance of AI-Based CMR Strain Analysis

Each case consisted of 3 standard views (2-chamber view, 3-chamber view and 4-chamber view), resulting in a total of 333 chamber views being evaluated by the artificial intelligence program. Among these, 20 (6.0%) required manual corrections due to inaccuracies in the AI’s interpretation of myocardial strain.

Error Distribution Across Chamber Views:

2-Chamber View: 2 cases required corrections.

3-Chamber View: 11 cases required corrections.

4-Chamber View: 7 cases required corrections.

**Figure 1:**
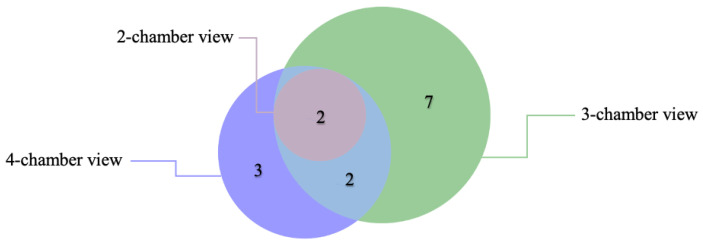
Error distribution and overlap across 2-chamber, 3-chamber, and 4-chamber views in AI-based CMR strain analysis among affected individuals.

Even though 20 chamber views required manual corrections, these involved 14 CMR cases, as shown in Figure 1. Overlapping errors were infrequent, 14.3% (2 cases) occurred in both the 3- and 4-chamber cine views, and another 14.3% (2 cases) spanned all three views. Among the remaining errors, 50% (7 cases) occurred exclusively in the 3-chamber cine view, making it the most error-prone, while 21.4% (3 cases) were detected only in the 4-chamber view. No standalone errors were detected in the 2-chamber view.

### 3.2. Comparison of the Performance of AI-Based CMR Feature Tracking Technique Between Aortic Stenosis and Healthy Control Groups

The overall error rate was substantially higher in the AS group. Manual corrections were required in at least one chamber view in 13 out of 70 patients (18.6%) within the AS group. In contrast, only 1 in 41 healthy controls (2.4%) required manual intervention. To assess the statistical significance of this observed difference, Fisher’s exact test was applied. The test revealed a significant disparity in error rates between the two groups (*p* = 0.0088), confirming that the AI system exhibited greater challenges in accurately interpreting myocardial strain in patients with AS.

### 3.3. Critical Limitation in AI Performance

**Figure 2:**
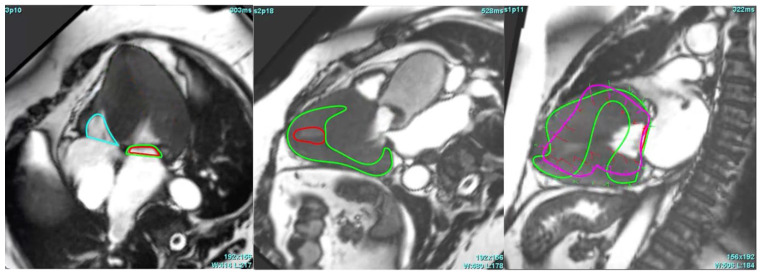
AI-based segmentation in CMR views of an aortic stenosis patient, illustrating challenges in marking the endocardium (red) and epicardium (green) in 4-, 3-, and 2-chamber views.

One case in the AS group, as shown in Figure 2, was entirely unanalysable by the AI. In this instance, the automated LV segmentation failed, and strain analysis was impossible until manual corrections were applied.

### 3.4. Segmental and Global AI-Based CMR Myocardial Strain Analysis in AS Patients vs. Controls

The most notable differences were identified in the basal segments (Table 1). The Basal Inferoseptal segment showed the greatest strain reduction, with AS patients averaging −20.23 (SD = 5.77) compared to −28.09 (SD = 3.58) in healthy controls (mean difference: 7.86, *p* < 0.001). The smallest myocardial strain reductions were observed in the Basal Anterolateral segment in AS patients vs. controls (−28.36, SD = 6.16 vs. −31.41, SD = 4.86, mean difference: 3.05, *p* = 0.009) and the Basal Anterior segment (−21.66, SD = 6.48 vs. −24.89, SD = 4.85, mean difference: 3.23, *p* = 0.004). All basal segments (100%) showed statistically significant differences in GLS between AS patients and healthy controls (*p* < 0.05).

The average reduction in GLS in middle segments was smaller, with greatest difference between AS patients and healthy cohort in the Middle Anterior (−14.05, SD = 5.11 vs. −18.65, SD = 5.10, mean difference: 4.60, *p* < 0.001) and Middle Inferior (−13.62, SD = 6.19 vs. −16.84, SD = 5.16, mean difference: 3.22, *p* = 0.007) segments. The smallest average GLS differences were seen in the Middle Anteroseptal (−16.46, SD = 6.09 vs. −16.23, SD = 4.63, mean difference: −0.23, *p* = 0.837) and Middle Inferoseptal (−14.50, SD = 6.31 vs. −13.64, SD = 5.52, mean difference: −0.87, *p* = 0.473) segments. In the middle segments, this difference was statistically significant in 33% (2 out of 6) of segments.

The apical segments generally exhibited bigger global longitudinal strain as compared with basal segments, with the most notable impairment observed in the Apical Lateral segment, where the average strain was significantly reduced in AS patients (−11.45, SD = 5.38) compared to controls (−14.92, SD = 5.68), resulting in a mean difference of 3.48, *p* = 0.002. A significant reduction was also observed in the Apex (−14.55, SD = 5.54 vs. −16.90, SD = 4.13, mean difference: 2.35, *p* = 0.013). All other apical segments (3 out of 5) showed small differences between AS patients and healthy controls, none of which were statistically significant.

AI-based GLS derived from CMR in AS patients was significantly reduced compared to controls (−17.86 ± 3.47 vs. −20.70 ± 1.98), indicating impaired myocardial deformation in AS. The higher standard deviation in AS patients indicates greater variability in strain measurements, likely due to heterogeneous myocardial remodelling and varying disease severity.

### 3.5. Comparison of AI-Based CMR GLS with Echocardiography-Based GLS in AS Patient Cohort

**Figure 3:**
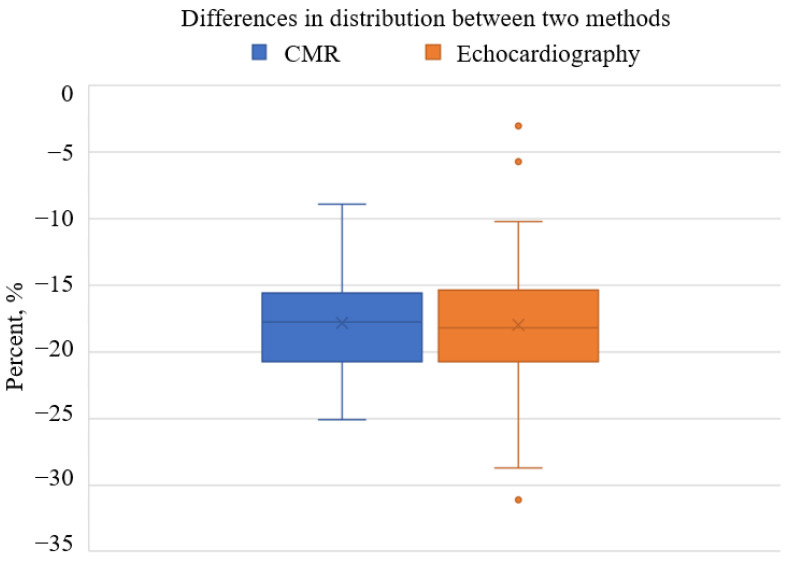
Boxplot Comparison of CMR and Echocardiography in Myocardial Strain Assessment.

CMR demonstrated a slightly higher mean myocardial strain value (−17.86, SD = 3.47) compared to echocardiography (−18.00, SD = 4.98) in the same AS patient cohort. The standard deviation for CMR was notably smaller (3.47 vs. 4.98). This indicates reduced variability in strain measurements across patients. This precision was illustrated by boxplot analysis (Figure 3). CMR showed a narrower interquartile range and fewer outliers, whereas echocardiography displayed greater variability with a wider interquartile range and some extreme outliers.

A strong concordance between the two methods was observed. It was supported by a significant positive correlation (r = 0.694, *p* < 0.001). The mean difference between CMR and echocardiography was −0.14, SD = 3.59, with a 95% confidence interval ranging from −0.71 to 0.99, indicating no systematic bias (*p* > 0.05). These findings suggest that differences between the two methods are attributable to random variability rather than inherent methodological discrepancies.

## 4. Discussion

This is a retrospective study that compared the automatic LV myocardial strain analysis of CMR images performed by the AI between an AS and healthy patients’ groups, as well as the strain analysis between echocardiography and CMR. Our results demonstrate that AI-based CMR strain analysis is a reliable tool that accurately found reduced GLS in the AS group compared to healthy controls, with the most pronounced differences in the basal LV segments. Additionally, we found a strong correlation between AI-based strain values and echocardiographic strain measurements, with CMR showing lower variability between the patients. Some CMR images required manual corrections of automatic segmentation done by the AI, most often in the 3-chamber view images, with the errors being most prominent in the AS patients’ group.

Speckle tracking echocardiography (STE) is the primary modality used for myocardial strain assessment in a variety of heart diseases [11]. It is a widely available method for GLS analysis that tracks the speckles of myocardium and their displacement [12]. In contrast, the CMR feature tracking technique relies on the deformation of endocardial and epicardial borders [13]. Compared to CMR, STE is a more accessible, faster, and less expensive approach with lower inter-manufacturer variability; however, its accuracy depends on adequate acoustic windows and may be limited in certain body types [14]. While STE is the most often used tool for GLS analysis, CMR feature tracking is an additional tool and may serve as a superior method in some cases due to echocardiographic limitations and good CMR’s reproducibility [7,15,16]. Relative apical sparing (RAS) is a strain distribution that has been observed for some patients with AS, where basal longitudinal strains are more reduced compared to the apical segments [10,17]. While RAS is only observed in about 15.3–18% of the AS patients, increased volume and pressure cause imbalanced LV wall stress, which leads to reduced longitudinal function, especially notable at the basal section of the LV [10,18,19]. Our study’s findings, based on AI-driven CMR feature tracking, corresponded with these findings, showing the most notable differences between healthy controls and the AS group at the basal LV segments (mean difference: 7.86, *p* < 0.001), with all basal segments exhibiting statistically significant GLS differences (*p* < 0.05). The difference was less pronounced in the middle and apex segments, with statistical significance observed only in the middle anterior (mean difference: 4.60, *p* < 0.001) and middle inferior segments (mean difference: 3.22, *p* = 0.007), as well as the apical lateral segment (mean difference: 3.48, *p* = 0.002) and the apex itself (mean difference: 2.35, *p* = 0.013). No statistically significant differences were found between the healthy patients and the AS cohort in any other segments. These findings support the imbalanced decrease of the function of different LV segments and also confirm the overall reduction of GLS in AS patients.

The analysis results of echocardiography and AI-based CMR feature tracking myocardial strain measurements showed a strong correlation between the methods (positive correlation: r = 0.694, *p* < 0.001). We also found the CMR method to have fewer variable results and no marked outliers. Other studies have previously explored these techniques and compared their measurements [15,16]. Good correlations were observed between the GLS and CMR feature tracking, as well as with CMR LV ejection fraction. However, the previous studies did not find enough evidence for interchangeable usage of both methods due to a wide limit of agreement or limited evidence in certain subgroups [15,20]. Although our findings also demonstrated strong correlations, the cohort size may have been too small and only included AS patients and healthy controls to definitively establish the equivalence of both modalities. Potential advantages of CMR feature tracking have been proposed, such as higher signal-to-noise ratio, good image reproducibility, poor acoustic windows of echocardiography and high framerate requirement for STE, while echocardiography benefits from faster obtainable images and is good at identifying speckles inside compact myocardium, which is difficult to track for CMR [21].

CMR feature tracking is usually a semiautomated process using a propagation method [20,22]. In our study, we used a fully automated AI-based CMR strain software, which is under continued review, in the program SuiteHeart Version 5.1.1 (Neosoft, Pewaukee, WI, USA). Overall, we found this automatic instrument to be relatively precise, with 6% of all views requiring manual correction, most often in the 3-chamber and 4-chamber views. However, mistakes were much more common for images of AS patients, compared to the control group (18.6% vs. 2.4%). The usual reason for these errors was a failed LV segmentation, usually caused by significant LV hypertrophy, common amongst AS patients, which explains why the health control had many fewer inconsistencies [23]. AI tools being less accurate in the pathology group in CMR feature tracking has been published before, particularly in LV hypertrophy patients [24]. Difficulties in contouring the myocardium, thicker left ventricle walls, and hypertrophied papillary muscles may prove challenging for automatic tools to correctly mark endocardial and epicardial contours, potentially leading to an overestimation of GLS [13,24]. Therefore, precise segmentation is essential. In our cohort, after the human corrections, only 1 out of 111 cases was unable to be segmented and analysed for GLS. Elevated values of STE due to apex foreshortening and focal myocardial bulging in patients with LV hypertrophy have also been reported. [14,25].

AI is an evolving technology increasingly used in all cardiac imaging modalities, including CMR, for more informative and accurate analysis [26]. AI is used in CMR to complete a variety of tasks. Biventricular function evaluation of cine CMR images is a time-consuming process that becomes faster using AI, and its semiautomated or fully automated analysis provides a lower inter-operator variability [27]. Wang et al. [28] implemented deep learning AI algorithms for left and right ventricle (RV) analysis. They found good correspondence between human measurements and AI results for LV ejection fraction (EF) and LV mass, while RV EF was less accurate. Another study integrated AI into CMR for detecting aortic stenosis. The model achieved impressive results with 95% recall and 96% precision in detecting aortic stenosis, even with limited data [29]. A study by Evertz et al. [30] analysed a similar population to our study with severe aortic stenosis patients undergoing transcatheter aortic valve replacement, assessed the prognostic value of fully-automated LV function evaluation, and showed strong correlation with a manual approach. Augustoet al. [31] found that machine learning based AI measures LV wall thickness better than experts in patients with hypertrophic cardiomyopathy. Many studies have recently explored fully automated CMR segmentation methods; however, there still remain some challenges, such as hindered identification of basal and apical myocardium, differentiation between trabeculae and myocardium, and difficulty generalising AI models [32]. These troubles are being addressed using more sophisticated AI algorithms [33,34]. There are promising results—for example, a study by Assadi et al. has shown good results not only for four-chamber segmentation, but also for evaluating patient prognosis [35]. Another important field of analysis is contrast-free CMR imaging. Usually, late gadolinium enhancement (LGE) is required to visualise myocardial scar, which develops due to several different causes, such as myocardial infarction, myocarditis, sarcoidosis, different cardiomyopathies, and others [36]. Various deep learning and machine learning techniques are being explored to achieve an equivalent visualisation of the scarring without using contrast [37]. Xu et al. [38] used a generative adversarial network, a type of machine learning AI, and achieved 96.98% accuracy for myocardial infarction area segmentation in contrast-free CMR images. Another study used deep learning methods and showed good correlation with both LGE images and histological samples [39]. Notable progress is underway in the area of radiomics—the process of extraction of quantitative metrics from medical images, which analyses the radiomic features of pixel distribution, shapes, texture and others [40]. This technique is also applicable to CMR and can augment the analysis of myocardial structure, find subtle changes of the myocardium, help differentiate between diseases, and have prognostic value [41]. Neisius et al. [42] used radiomics to distinguish patients with LV hypertrophy due to hypertensive heart disease and hypertrophic cardiomyopathy in T1 native maps. This method was also found to be feasible for improved CMR stress perfusion [43], detecting myocardial scar [44,45], identifying fibrosis and inflammation in patients with dilated cardiomyopathy [46], and cardiac sarcoidosis [47].

The older versions of the program Suiteheart, which we used in our study, have also been applied in several other publications, some of which had certain similarities to our work. Zhang et al. [48] compared three separate software packages, including Suiteheart. The systems were able to differentiate between healthy controls and patients with preserved EF heart failure, while Suiteheart’s gradient echo cine 4-chamber GLS correlated with outcomes. A forementioned study by Evertz et al. [30] also used this software for the analysis of patients with AS. A similar percentage of images required manual corrections compared to our study’s AS group (15.8% vs. 18.6%). About 10 min of time saved per patient using AI was emphasised in the article. Chudgar et al. further explored GLS analysis using this program [49]. Other articles have described using it for automatic segmentation for paediatric patients with or without congenital heart diseases [50], assessing biventricular function after cardiac resynchronisation therapy [51], separating structural changes due to cardiac amyloidosis and hypertensive patients with heart failure [52], and predicting cardiovascular outcomes for HIV patients according to myocardial fibrosis and inflammation. Backhaus et al. [53] used it for LV and RV volume and function evaluation and found the algorithm to require fewer manual corrections for LV compared to RV.

Our study is another addition to the ocean of AI models that are being used for CMR and other imaging modalities. We tested a new AI tool, which is under continued review, and showed its viability for CMR strain analysis, which was comparable to echocardiography GLS. The strain pattern was typical for AS patients, and almost all images could be analysed automatically or with minimal manual corrections. The percentage of required adjustments was similar to previously published studies.

### Limitations

This is a small retrospective single-centre study that only included patients with AS and healthy controls, so the results might not be applicable to other populations. Also, our study did not compare the outcomes of patients relying on GLS results. Larger studies are required to evaluate the AI program’s performance in other cardiac diseases as well as its prognostic value. Some of the images required manual corrections, and investigator bias may have altered the results. One patient could not be included in the analysis. This highlights the need for good-quality images to be able to apply AI tools for the assessment. It is also noteworthy that CMR is a more expensive and less accessible imaging modality worldwide compared with echocardiography. Although the AI-based CMR feature tracking technique is a feasible option to assess myocardial strain in all patients undergoing scans, its wide adoption into clinical practice may be limited due to the low availability and high cost of CMR scanners. However, a one-stop shop assessment by using all advancements of CMR techniques, including feature tracking, allows not only a more comprehensive assessment of cardiac morphology, function and structure, but also allows for assessing suitable changes at follow-up.

## 5. Conclusions

AI-based CMR shows high accuracy and precision for GLS measurements, good agreement with the reference standard of echocardiography and reduced variability. CMR performed well in strain analysis but had a 6% error rate. Errors were much higher in AS patients (18.6%) than in healthy controls (2.4%). Segmental strain deficits were greatest in basal myocardial segments in AS patients, where reduced strain was less pronounced in middle and apical segments. Benefits of CMR in identifying subtle GLS impairments and the potential for automated, consistent, reproducible measurements are emphasised, but further refinement of AI algorithms to solve challenges in severe pathology and facilitate the establishment of reference standards is also encouraged.

## Figures and Tables

**Table 1:** Mean GLS in Cardiac Segments Between Aortic Stenosis Patients and Healthy Controls.

Group Statistics and Independent Samples *T*-Test for Equality of Means: Mean GLS in Cardiac Segments Between Aortic Stenosis Patients and Healthy Controls						
	Aortic Stenosis	N	Mean	Mean Difference	Std, Deviation	Two-Sided *p*
Basal Anterior	No	40	−24.89	3.23	4.85	0.004
	Yes	68	−21.66		6.48	
Basal Anteroseptal	No	40	−21.03	6.11	5.40	<0.001
	Yes	68	−14.92		5.83	
Basal Inferoseptal	No	40	−28.09	7.86	3.58	<0.001
	Yes	68	−20.23		5.77	
Basal Inferior	No	40	−36.12	6.41	3.73	<0.001
	Yes	68	−29.72		5.05	
Basal Inferolateral	No	40	−36.10	6.20	3.51	<0.001
	Yes	68	−29.90		5.69	
Basal Anterolateral	No	40	−31.41	3.05	4.86	0.009
	Yes	68	−28.36		6.16	
Mid Anterior	No	40	−18.65	4.60	5.10	<0.001
	Yes	68	−14.05		5.11	
Mid Anteroseptal	No	40	−16.23	−0.23	4.63	0.837
	Yes	68	−16.46		6.09	
Mid Inferoseptal	No	40	−13.64	−0.87	5.52	0.473
	Yes	68	−14.50		6.31	
Mid Inferior	No	40	−16.84	3.22	5.16	0.007
	Yes	68	−13.62		6.19	
Mid Inferolateral	No	40	−15.35	1.09	5.36	0.385
	Yes	68	−14.26		6.75	
Mid Anterolateral	No	40	−19.29	2.29	5.38	0.079
	Yes	68	−17.00		7.04	
Apical Anterior	No	40	−15.08	−0.64	5.04	0.605
	Yes	68	−15.72		6.81	
Apical Septal	No	40	−16.79	0.57	4.34	0.592
	Yes	70	−16.23		6.69	
Apical Inferior	No	40	−10.59	−0.96	6.27	0.423
	Yes	68	−11.55		5.80	
Apical Lateral	No	40	−14.92	3.48	5.68	0.002
	Yes	70	−11.45		5.38	
Apex	No	40	−16.90	2.35	4.13	0.013
	Yes	70	−14.55		5.54	

## Data Availability

The original contributions presented in this study are included in the article. Further inquiries can be directed to the corresponding author(s).
